# Tissue‐Resident Memory CD8+ T Cells: Differentiation, Phenotypic Heterogeneity, Biological Function, Disease, and Therapy

**DOI:** 10.1002/mco2.70132

**Published:** 2025-03-10

**Authors:** Luming Xu, Lilin Ye, Qizhao Huang

**Affiliations:** ^1^ Provincial Key Laboratory of Immune Regulation and Immunotherapy, School of Laboratory Medicine and Biotechnology Southern Medical University Guangzhou China; ^2^ Institute of Immunology Third Military Medical University Chongqing China; ^3^ Institute of Immunological Innovation and Translation Chongqing Medical University Chongqing China

**Keywords:** autoimmune diseases, CD8^+^T_RM_, ICB therapy, infection, tumor, vaccine

## Abstract

CD8+ tissue‐resident memory T cells (TRM) are strategically located in peripheral tissues, enabling a rapid response to local infections, which is different from circulating memory CD8+ T cells. Their unique positioning makes them promising targets for vaccines designed to enhance protection at barrier sites and other organs. Recent studies have shown a correlation between CD8+ TRM cells and favorable clinical outcomes in various types of cancer, indicating their potential role in immune checkpoint blockade (ICB) therapies. However, the dual nature of CD8+ TRM cells presents challenges, as their inappropriate activation may lead to autoimmunity and chronic inflammatory conditions. This review highlights significant advancements in the field, focusing on the differentiation pathways and phenotypic heterogeneity of CD8+ TRM cells across different tissues and disease states. We also review their protective roles in various contexts and the implications for vaccine development against infections and treatment strategies for tumors. Overall, this comprehensive review outlines the common features of CD8+ TRM cell differentiation and biological functions, emphasizing their specific characteristics across diverse tissues and disease states, which can guide the design of therapies against infections and tumors while minimizing the risk of autoimmune diseases.

## Graphical abstract

Xu et al. described the differentiation, biological function, and phenotype heterogeneity of CD8^+^T_RM_ cells across various types of tissues and distinct disease settings, and discussed their potential roles in the designation of vaccines against infectious diseases and immune therapy of tumors

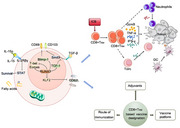

## 1 Introduction

Memory CD8^+^ T cells play a crucial role in protecting against reinfection by intracellular pathogens and in monitoring tumor progression [1]. Traditionally, two distinct populations of memory CD8^+^T cells have been characterized: effector memory CD8^+^T (CD8^+^T_EM_) cells and central memory CD8^+^T (CD8^+^T_CM_) cells [2]. CD8^+^T_EM_ cells are a subset of memory T lymphocytes that mount rapid immune responses upon re‐exposure to pathogens. These cells circulate between the bloodstream and nonlymphoid tissues (NLTs) due to their lack of secondary lymphoid organ (SLO)‐homing receptors, such as CD62L (L‐selectin) and CCR7 [2]. In contrast, CD8^+^T_CM_ cells express CD62L and are enriched in SLOs, where they retain the capacity to proliferate and generate effector cells during antigen rechallenge [2]. Subsequent studies have identified a distinct subset—tissue‐resident memory CD8^+^T cells (CD8^+^T_RM_)—which permanently reside in NLTs without recirculating. For instance, memory CD8^+^T cells generated in the dorsal root ganglia and skin following herpes simplex virus (HSV) infection [3], as well as those in the small intestine after lymphocytic choriomeningitis virus (LCMV) infection [4], were shown to remain in their respective tissues long after the resolution of infection, without re‐entering the bloodstream.

The presence of CD8^+^T_RM_ cells has been confirmed across various tissues, including the epithelium of the lung [5] and the female reproductive tract [6], as well as the parenchyma of salivary glands [7], kidneys [8], brain [9], and thymus [10]. Notably, CD8^+^T_RM_ cells have also been identified in the subcapsular sinus of lymph nodes and the marginal zone of the spleen [11], and within the sinusoids of the liver [12, 13]. These findings highlight the widespread distribution of CD8^+^T_RM_ cells in peripheral tissues, where they establish a permanent presence after infections are resolved. This residency enables CD8^+^T_RM_ cells to act as the first line of defense against reinfection, allowing for rapid responses to local pathogen re‐exposure [14]. The rapid response of CD8^+^T_RM_ cells contrasts with the activity of circulating memory CD8^+^ T cells(T_CIRCM_), including T_CM_ and effector memory (T_EM_) subsets, which typically require time to migrate from the bloodstream to the site of infection [14]. Given their strategic role in providing immediate protection at barrier organs, the efficient induction of CD8^+^T_RM_ cells has emerged as a promising strategy for vaccine design [15].

In addition to infection‐induced CD8^+^T_RM_ cells, vaccination and tumor inoculation can also lead to the generation of CD8^+^T_RM_ cells, which are vital for effective cancer immune surveillance [16, 17]. Both CD8^+^T_RM_ and CD8^+^T_RM_‐like cells have been identified in various solid tumors and are often associated with improved clinical outcomes [18, 19, 20, 21, 22, 23]. However, it is noteworthy that CD8^+^T_RM_ and CD8^+^T_RM_‐like cells within progressive tumors tend to exhibit dysfunctional characteristics, marked by the upregulation of exhaustion markers [20, 23–28]. This dysfunction presents a unique opportunity to target these cells for immune checkpoint blockade (ICB) therapy. Emerging evidence suggests that CD8^+^T_RM_ and CD8^+^T_RM_‐like cells, which are distinct from CD8^+^ progenitor exhausted T (Tpex) cells, can directly respond to ICB treatment in clinical settings, paving the way for enhanced efficacy of ICB therapies [27, 29–31]. ICB strategies are designed to reinvigorate exhausted T cells infiltrating tumors by blocking inhibitory molecules such as PD‐1/PD‐L1 and CTLA‐4 [2]. While prior studies have established Tpex cells as primary responders to ICB [32, 33, 34], demonstrating their role in proliferative expansion and differentiation into more effector‐like exhausted CD8^+^T (Tex) cells [35], recent findings have highlighted the importance of tumor‐specific stem‐like memory CD8^+^T (Ttsm) cells in tumor‐draining lymph nodes (TdLNs) as also being bona fide responders to ICB [36]. Given that the objective response rates to ICB therapy remain relatively low across many solid tumors, the recognition of CD8^+^T_RM_ and CD8^+^T_RM_‐like cells as potential novel responders to ICB therapy offers a promising avenue to enhance the efficacy of immune interventions.

While the induction of CD8^+^T_RM_ cells presents promising opportunities for designing effective vaccines against infections and tumors, as well as enhancing the efficacy of immunotherapy, it is essential to recognize the potential downsides of their presence. Specifically, CD8^+^T_RM_ cells can contribute to the development of autoimmune diseases and chronic inflammatory disorders, leading to their characterization as a “double‐edged sword” in immunological contexts [37, 38]. Therefore, understanding the regulatory mechanisms governing CD8^+^T_RM_ cell activity is crucial for harnessing their benefits while mitigating adverse effects. CD8^+^T_RM_ cells residing in various tissues exhibit common characteristics, such as heightened expression of retention molecules and the downregulation of egress markers, which facilitate their long‐term residence in peripheral tissues. However, the mechanisms that drive the formation and maintenance of these cells can differ significantly depending on the tissue type and the specific disease context [39, 40, 41]. This variability underscores the need for tailored therapeutic strategies that consider the unique regulatory pathways of CD8^+^T_RM_ cells in different tissue sites and disease states. Clarifying how CD8^+^T_RM_ cell activity is regulated will not only enhance our understanding of their roles in immunity but also inform the design of targeted therapies that maximize their protective functions while minimizing the risk of autoimmune complications.

As compared with previous reviews, which specifically focus on CD8^+^T_RM_ cells differentiation, and phenotype heterogeneity [14, 42, 43], or their roles in antitumor immunity [44, 45] or anti‐infection immunity [15, 46], in this review, we comprehensively examined the advancements in our understanding of the differentiation of CD8^+^T_RM_ cells and their phenotypic heterogeneity across various tissues and disease contexts. Furthermore, we explored the function of CD8^+^T_RM_ cells, and their relationship with various diseases, including infections, cancer, and autoimmune disorders. Finally, we considered the potential applications of CD8^+^T_RM_ cells in immunotherapy, particularly in the design of vaccines and therapeutic strategies aimed at enhancing immune responses while minimizing adverse effects.

## 2 Differentiation and Phenotype Heterogeneity of Cd8^+^T_RM_ Cells

Two primary models have been proposed to explain the mechanisms underlying the differentiation of CD8^+^T_RM_ cells: One is the “local divergent” model, in which the decision for CD8^+^T_RM_ lineage commitment occurs within the tissue itself. The other is the “systematic divergent” model, in which the lineage decision for CD8^+^T_RM_ cells is made during the initial activation phase, likely in SLOs. Subsequently, the local tissue microenvironment influences the generation and maintenance of these cells once they migrate to peripheral tissues. Current research predominantly supports the “systematic divergent” model for the differentiation of CD8^+^T_RM_ cells [47]. Under this model, the formation and persistence of CD8^+^T_RM_ cells are heavily influenced by the local tissue microenvironment.

### 2.1 Formation and Maintenance of CD8^+^T_RM_ Cells During Infections

**FIGURE 1:**
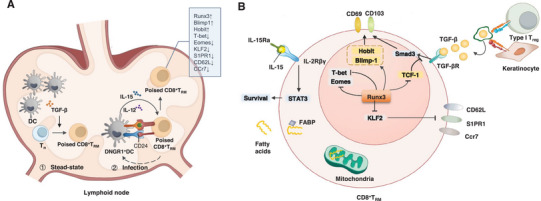
The commitment of CD8^+^T_RM_ in lymphoid tissues and their differentiation in specific tissue. (A) The precursors of CD8^+^T_RM_ are poised in lymphoid tissues under two conditions. Under normal physiological conditions, CD8^+^T_RM_ cells are developed from naïve CD8^+^T cells in the presence of TGF‐β activated by migratory DCs. Upon infection, naïve CD8^+^T cells undergo cross‐priming by DNGR1^+^DCs. Meanwhile, DCs secrete key cytokines, including IL‐15 and IL‐12, and upregulate costimulatory molecules CD24 during this activation process. The integration of these signals—IL‐15, IL‐12, and costimulatory signals—ensures a robust differentiation of CD8^+^T cells into CD8^+^T_RM_ cells. Significant changes of transcription factors (TFs) expression occurs in the committed CD8^+^T_RM_ cells, such as upregulation of Runx3, Blimp1, and Hobit, but downregulation of T‐bet, Eomes, and Krüppel‐like factor 2 (KLF2). (B) After enter into tissues, multiple TFs work in concert to promote the retention of CD8^+^T_RM_ precursors in the tumor. Runx3, a central transcription factor for CD8^+^T_RM_ cells, promotes the expression of key factors such as Blimp1 and Hobit. This results in the upregulation of important tissue‐residency molecules, including CD103 and CD69. Concurrently, Runx3 suppresses the expression of T‐bet, Eomes, and KLF2, which leads to the downregulation of tissue‐egress molecules such as S1PR1, CD62L, and CCR7. Additionally, the formation and maintenance of CD8^+^T_RM_ cells in tissues are regulated by inflammatory cytokines and nutrient availability. For instance, TGF‐β, which is activated by integrin (αvβ6 and αvβ8) on Type 1 Treg cells or keratinocytes, promotes the formation of CD8^+^T_RM_ by upregulating CD103 and CD69 expression. IL‐15, transpresented by DCs, may further enhance T_RM_ generation in the tissue. Moreover, exogenous fatty acids transported into T_RM_ cells by fatty acid‐binding protein (FABP) are essential for the long‐term survival of CD8^+^T_RM_ cells in tissues by providing necessary nutrients and energy.

The prevailing evidence suggests that CD8^+^T_RM_ cells primarily originate from effector CD8^+^T cells [48, 49, 50]. This has been supported by studies examining T cell receptor (TCR) clonality, where high‐throughput sequencing of the TCRβ gene revealed that both CD8^+^T_RM_ cells and T_CIRCM_ cells share common TCR clones [51]. Additionally, another study utilized lineage‐tracing and single‐cell transcriptome analysis to demonstrate that different TCR clones have varying propensities to give rise to CD8^+^T_RM_ or CD8^+^T_CIRCM_ precursor cells [52]. Despite some apparent contradictions, both studies converge on the idea that the lineage commitment toward CD8^+^T_RM_ or CD8^+^T_CIRCM_ occurs in circulating effector CD8^+^T cells before they migrate into tissues. However, an emerging perspective indicates that the potential for CD8^+^T_RM_ commitment may also be established at the naïve CD8^+^T cell stage under steady‐state conditions [53]. Upon infection, specific dendritic cell (DC) subsets also play critical roles in guiding the early differentiation of CD8^+^T_RM_ cells. For example, DNGR1^+^DCs are essential for the optimal priming of CD8^+^T_RM_ cells in skin and lung tissues in mice, while CD11b^+^DCs do not fulfill this role as effectively [54]. In humans, lung‐tissue‐resident CD1c^+^DCs, rather than CD141^+^DCs, preferentially induce the formation of CD8^+^T_RM_ cells in lung epithelia, and this process is dependent on the presence of the membrane‐bound cytokine transforming growth factor beta (TGF‐β) [55] (Figure 1A).

The differentiation of naïve CD8^+^T cells into CD8^+^T_RM_ cells is influenced by signals from DC subtypes, which promote two key biological properties. First, DCs shape the epigenetic and transcriptional landscape necessary for CD8^+^T_RM_ cell lineage decisions. Specific transcription factors (TFs) play crucial roles in this process. For instance, T‐bet (*Tbx21*), Eomes (*Eomesodermin*), TCF1 (*Tcf7*), and Krüppel‐like factor 2 (KLF2) are significantly suppressed in poised CD8^+^T_RM_ cells [50, 56–59]. High expression levels of T‐bet and Eomes negatively regulate TGF‐β receptor expression, which is critical for CD8^+^T_RM_ cell formation [56, 60, 61]. TCF1 inhibits the formation of lung CD8^+^T_RM_ cells by suppressing TGF‐β‐induced CD103 expression [62]. Additionally, the lack of KLF2 promotes the establishment of CD8^+^T_RM_ by reduces the expression of its target gene *S1pr1* (which encodes S1P1, sphingosine‐1‐phosphate receptor), leading to diminished S1PR1 signaling and the associated egress of T cells from lymphoid tissues [59]. Conversely, certain transcriptional regulators, including runt‐related TF (Runx) 3, B lymphocyte‐induced maturation protein 1 (Blimp1), the homolog of Blimp‐1 (Hobit), Basic Helix‐Loop‐Helix Family Member E40 (Bhlhe40), and Nuclear Receptor Subfamily 4 Group A Member 1 (Nr4a1), enhance CD8^+^T_RM_ cell formation. Runx3 is essential for establishing CD8^+^T_RM_ populations across diverse tissues [16]. Blimp1 and Hobit are specifically upregulated in CD8^+^T_RM_ cells and promote their formation by inhibiting the expression of proteins involved in tissue egress [63]. Genetic deletions of Bhlhe0 and Nr4a1 have been shown to selectively impair CD8^+^T_RM_ cell formation in mice [64, 65]. Second, DC subsets that commit CD8^+^T cells to the CD8^+^T_RM_ lineage enhance their capacity to accumulate in tissues. This is achieved by increasing tissue entry and retention through the upregulation of chemokine receptors like CXCR3 and CXCR6. These receptors are associated with the enhanced ability of committed CD8^+^T_RM_ cells to migrate into tissues, particularly in the skin and respiratory airways, facilitating the formation of CD8^+^T_RM_ cells in these locations [50, 66]. Overall, the interplay between DC signaling and transcriptional regulation is crucial for the successful differentiation of CD8^+^T_RM_ cells (Figure 1A).

Other signals in lymphoid tissues help guide T cells toward the T_RM_ cell lineage by regulating either of the above biological properties, with TGF‐β being one of the most significant factors. TGF‐β plays a crucial role in directing CD8^+^T_RM_ cell differentiation both during steady‐state conditions and infection. In the absence of foreign antigens, TGF‐β activation by migratory DCs in the lymph nodes enhances the epigenetic accessibility of genes associated with the CD8^+^T_RM_ cell signature [53]. During an infection, CD8^+^T cells initially activate and rapidly downregulate TGF‐β receptor expression. However, they regain TGF‐β receptor expression approximately 24 h after activation [67, 68]. This temporal regulation may be influenced by P2RX7, an extracellular receptor that responds to adenosine triphosphate (ATP). In the absence of P2RX7, splenic T cells exhibit reduced expression of *Itgae* (the gene encoding CD103) and elevated levels of *Eomes*, leading to diminished CD8^+^T_RM_ cell formation [68] (Figure 1A).

During the cross‐priming of naïve CD8^+^T cells, DNGR1^+^ DCs play a pivotal role by secreting key cytokines such as IL‐15 and IL‐12, along with elevating the expression of the costimulatory molecule CD24. These three signals are essential for the differentiation of CD8^+^T_RM_ cells in both skin and lung tissues, although the exact mechanisms by which they influence CD8^+^T_RM_ differentiation remain to be fully elucidated [54]. IL‐12 is known to induce the expression of CD49a, which is critical for persistence and function of CD8^+^T_RM_ cells within skin and lungs [69, 70, 71]. Furthermore, both IL‐12 and IL‐15 are implicated in the activation of the mTORC1 (mammalian target of rapamycin complex 1) signaling pathway [72, 73]. The mTORC1 pathway is crucial for regulating T cell metabolism and growth, and the inhibition of mTORC1 activity during T cell priming has been shown to significantly reduce CD8^+^T_RM_ cell formation. This indicates that proper mTORC1 signaling is necessary for T cells to commit to the CD8^+^T_RM_ lineage [73, 74, 75] (Figure 1A).

After entry into tissues, the formation and maintenance of CD8^+^T_RM_ cells are influenced by a variety of tissue‐specific factors that enable these cells to adapt to their local environments [39, 40]. Tissue‐specific factors predominantly include local antigen expression and inflammation. The presence of local antigens plays a crucial role in the formation of CD8^+^T_RM_ cells in specific tissues. For instance, local antigen presentation is necessary for the development of CD8^+^T_RM_ cells in the brain [9] and is also key in the liver [76], lung [77], and large intestine [78]. However, in other tissues such as the skin, female reproductive tract [79], intestinal epithelium [8], and upper respiratory tract [80], persistent local antigen stimulation is not required for the formation of CD8^+^T_RM_ cells. This highlights the variability in how different tissues regulate CD8^+^T_RM_ cell differentiation in response to antigens. In epithelial tissues like the skin, gut, and lung, the formation of CD8^+^T_RM_ cells is also predominantly regulated by the cytokine TGF‐β [8, 49, 50, 81]. TGF‐β is secreted in an inactive form bound to latency‐associated peptides (LAPs) and is activated by integrins such as αvβ6 and αvβ8, which facilitate the dissociation of LAPs [82]. In the epidermis, TGF‐β activation is specifically mediated by keratinocyte‐expressed integrins. This activation is crucial for promoting both the formation and maintenance of CD8^+^T_RM_ cells in the skin [83, 84]. Moreover, the transactivation of autocrine TGF‐β has been shown to enrich antigen‐specific CD8^+^T_RM_ cells within the epidermal niche [85]. TGF‐β activates TF Smad3 to induce the expression of CD103(α chain of the integrin αEβ7) [62], which is important for the persistence of CD8^+^T_RM_ cells in epithelial tissues [43], as it interacts with E‐cadherin on epithelial cells [86], for anchoring CD8^+^T_RM_ cells within the tissue. Interestingly, TGF‐β has also been shown to be required for the retention of intestinal CD8^+^T_RM_ cells partially through the induction of CD69 [87], which is essential for CD8^+^T_RM_ cells generation in kidney through binding to egress factor S1PR1 [88]. In contrast to epithelial tissues where TGF‐β plays a critical role in the formation and maintenance of CD8^+^T_RM_ cells, its involvement is not required for the generation of these cells in several other tissues [40], including the liver, adipose tissue, upper respiratory tract, and kidney. For instance, in the liver, the retention of CD8^+^T_RM_ cells occurs primarily through the interaction between Lymphocyte Function‐Associated Antigen 1 on CD8^+^T_RM_ cells and Intercellular Adhesion Molecule 1 on the endothelial cells lining the hepatic sinusoids. This interaction facilitates the anchoring of T_RM_ cells within the liver's unique microenvironment, allowing them to remain poised for rapid responses to hepatic pathogens without reliance on TGF‐β [89] (Figure 1B).

IL‐15 is another crucial cytokine for the formation of CD8^+^T_RM_ cells in various organs, including the skin, small intestine, liver, and kidney. It plays a significant role in promoting the survival, proliferation, and differentiation of these CD8^+^T_RM_ cells, ensuring their presence in tissues where they can respond to local infections [50, 72, 76, 90, 91], while IL‐15‐independent maintenance of CD8^+^T_RM_ cells has also been reported [92]. Other cytokines such as IL‐7, IL‐33, and tumor necrosis factor‐alpha (TNF‐α) have also been implicated in the regulation of CD8^+^T_RM_ cell formation [59, 93], but type I interferon (IFN) severe combined immune deficiencys can have an inhibitory effect, particularly in the liver [94]. For instance, their presence can limit the generation of liver CD8^+^T_RM_ cells following immunization with attenuated sporozoites [94]. However, the specific roles and mechanisms by which these cytokines influence CD8^+^T_RM_ development in different organs remain to be fully elucidated. Apart from cytokines, various immune cells can modulate the formation of CD8^+^T_RM_ cells. For example, CD4^+^T helper cells have been shown to promote the development of CD8^+^T_RM_ cells in the lung during influenza virus (IAV) infection. They achieve this through the reduction of T‐bet expression and secretion of IFN‐γ, which enhance the recruitment of circulating CD8^+^T cells into tissue and their differentiation of into CD8^+^T_RM_ cells [61]. Tissue‐resident CD4^+^T helper cells play a supportive role in generating CD8^+^T_RM_ cells, potentially through the secretion of cytokines like IL‐21, which can promote the survival and function of CD8^+^T cells [95]. Type 1 Treg cells have also been implicated in the generation of CD8^+^T_RM_ cells. They may exert their effects through the transactivation of TGF‐β, further illustrating the interplay between different T cell subsets in shaping the T_RM_ cell population [96]. The differentiation of CD8^+^T cells into CD8^+^T_RM_ cells also requires specific nutrients, particularly glucose and lipids, to fuel mitochondrial ATP production. Fatty acid‐binding protein (FABP) is critical for the transportation of exogenous lipids into CD8^+^T_RM_ cells [97], facilitating their long‐term survival in the microenvironment of NLTs. Different isoforms of FABP are utilized by various CD8^+^T_RM_ cells to adapt to the specific lipid composition present in their environment [98] (Figure 1B).

In summary, current researches support “the systematic divergent” model for the differentiation of CD8^+^T_RM_ cells. In this model, the activation of effector CD8^+^T cells at SLOs are poised to CD8^+^T_RM_ precursors, and the transformation of CD8^+^T_RM_ precursors into mature CD8^+^T_RM_ cells is dependent on tissue‐specific signaling. Therefore, although CD8^+^T_RM_ cells share common features such as reduced expression of tissue‐egress molecules and increased expression of tissue‐retention molecules, substantial differences exist depending on their tissue of origin [39, 40].

### 2.2 CD8^+^T_RM_ Generated and Maintenance in Tumors

The formation of CD8^+^T_RM_ cells, particularly in the context of vaccination or tumor inoculation, involves a complex interplay of precursor cells, DC subsets, TFs, and tissue–environment interactions. CD8^+^T_RM_ cells, whether induced by vaccination or in response to tumors, are derived from a population of Ttsm. This highlights the ability of these precursors to differentiate into CD8^+^T_RM_ cells during priming in TdLNs [41, 99]. While the specific DC subsets involved in CD8^+^T_RM_ cell development in mice remain undefined [100], there is evidence that certain subsets of human DCs, particularly CD1c^+^CD163^+^(DC3s), infiltrate tumors and have the capacity to induce differentiation of CD103^+^CD8^+^T cells in vitro [101, 102, 103, 104, 105]. After the initial priming of CD8^+^T cells, various TFs facilitate the differentiation of CD8^+^T_RM_ precursors and their retention in tumor tissues. Runx3, a central regulator for the development of CD8^+^T_RM_ cells across various tissues, is also upregulated in tumor‐specific CD8^+^T_RM_ cells [16]. Its upregulation in tumor‐specific CD8^+^T_RM_ cells promotes the expression of other important TFs, including Blimp1 and its homologue Hobit [63], and residency‐associated molecules, such as CD103 and CD69 [50, 88]. Meanwhile, Runx3 suppresses the expression of T‐bet [56] and Eomes, as well as KLF2, and downregulates tissue‐egress molecules including S1PR1, CD62L and CCR7 [59], further facilitating the retention of CD8^+^T_RM_ cells within the tumor microenvironment (TME).

The migration of CD8^+^T_RM_ cells within tumor tissues are crucial for establishing effective antitumor immune responses. The recruitment of committed CD8^+^T_RM_ precursors in various tissues are mediated by specific chemokine and chemokine receptor pairs. CXCR6 is a chemokine receptor expressed on intratumoral CD8^+^CD103^+^T_RM_ cells, and its absence leads to significant defects in the recruitment and retention of CD8^+^T_RM_ cells, resulting in diminished antitumor immune responses [106, 107, 108]. The retention of CXCR6^+^CD8^+^T_RM_ cells has been reported to be mediated by CXCR16 secreted by breast cancer cells, and the deficiency of CXCR6 promotes the egress of CD8^+^T_RM_ cells out of tumor tissues [109]. CXCL16 is also expressed by DCs and the interaction of which with CXCR6‐expressing CD8^+^T_RM_ cells is critical for the migration and persistence of CD8^+^T_RM_ cells and associated with melanoma protection [110].

After migrating into tumors, the formation of CD8^+^T_RM_ cells in various tissues is influenced by specific factors associated with the tissue microenvironment [39, 40]. Among these factors, TGF‐β, a critical factor in the formation of epithelial CD8^+^T_RM_ [50], is also necessary for the differentiation of stem‐like CD8^+^T cells into CD8^+^T_RM_ in TdLNs [99]. Additionally, the composition of the microbiota can impact CD8^+^T_RM_ cells generation and gastric cancer progression by modulating TGF‐β levels [111]. While direct evidence supporting the role of IL‐15 in promoting the formation of CD8^+^T_RM_ cells in tumors is still limited, it is known that IL‐15, when transpresented by DCs, enhances the survival of CD8^+^ cytotoxic T cells [112]. This suggests that while IL‐15 may not directly induce CD8^+^T_RM_ formation, it plays a supportive role in the recruitment and survival of effector T cells within the TME.

The formation and maintenance of CD8^+^T_RM_ cells within the TME are significantly influenced by metabolic factors, including low oxygen tension and nutrient availability. In response to hypoxia, hypoxia‐inducible factor (HIF)‐α is constitutively activated and drives the formation of CD8^+^T_RM_ cells in solid tumors [113], indicating a metabolic adaptation mechanism that supports CD8^+^T_RM_ cell differentiation and maintenance under hypoxic conditions. Additionally, a study has shown that providing additional fatty acids can invigorate tumor‐resident memory T cells, enhancing their survival and possibly their function within tumors [114]. Mitochondrial respiration components, such as coenzyme Q, are also found to be critical for the residency of CD8^+^T cells in tissues, and enhancing the synthesis of coenzyme Q has been linked to improved retention of CD8^+^T_RM_ cells in tumors, thus promoting antitumor immunity [115]. These findings highlight the importance of lipid metabolism in CD8^+^T_RM_ cell function in tumors.

**FIGURE 2:**
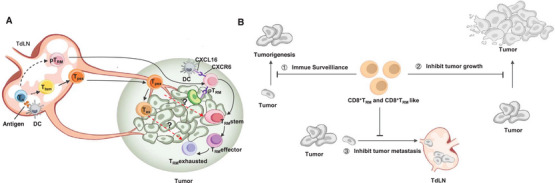
The differentiation of CD8^+^T_RM_ cells in tumors and their roles in antitumor immunity. (A) DCs capture tumor‐associated antigens and migrate to the tumor‐draining lymph nodes, where they prime CD8^+^T_RM_ precursors (pT_RM_) and Ttsm. The pT_RM_ cells are then recruited to the tumor microenvironment, where they differentiate into CD8^+^T_RM_ stem cells. This process is mediated by the interaction between CXCR6 on pT_RM_ cells and CXCL16 on DCs or cancer cells. As the tumor progresses, T_RM_ stem cells differentiate into two subsets: effector T_RM_ (CD8^+^T_RM_ effector) and exhausted T_RM_ (CD8^+^T_RM_ exhausted). Alternatively, Ttsm‐differentiated Tpex and Tex cells may acquire residency markers and become CD8^+^T_RM_ cells within the tumor microenvironment. (B) CD8^+^T_RM_ cells are essential players in the immune landscape against tumors, providing a first line of defense against tumorigenesis, actively inhibiting tumor growth, and preventing metastasis. The symbol “?” indicates the unknown relationships between these compartments.

Although both Runx2 and Runx3 activity are required to promote the differentiation of cytotoxic CD8^+^CD103^+^CD49a^+^T_RM_ cells, providing immunosurveillance of infected and malignant cells [116], emerging research suggests that tumor‐specific CD8^+^T_RM_ cells exhibit a residency profile that diverges from traditional markers associated with CD8^+^T_RM_ cells, such as CD49a and CD69. Instead, these tumor‐specific cells often express higher levels of exhaustion markers, including Tim‐3 and CD39 [41]. This phenomenon implies that the chronic antigen stimulation and unique characteristics of the TME alter the residency programs of these tumor antigen‐specific CD8^+^T_RM_ cells, leading to a distinct functional state compared to CD8^+^T_RM_ cells found in infections. These studies indicate that the differentiation pathway of tumor‐specific CD8^+^T_RM_ cells diverges from those of CD8^+^T_RM_ cells generated during infections, raising important questions about the mechanisms of CD8^+^T_RM_ cell persistence and functionality in tumors, which may impact therapeutic strategies aimed at enhancing antitumor immunity (Figure 2A).

### 2.3 Phenotype Heterogeneity of CD8^+^T_RM_ Cells

CD8^+^T_RM_ cells exhibit significant heterogeneity in their transcriptional and epigenetic profiles, influenced by the unique microenvironments of various tissues. For example, the canonical T_RM_ marker CD103 is prominently expressed in CD8^+^ T cells from epithelial tissues like the skin, small intestine, and salivary glands. In contrast, CD103 is absent in CD8^+^T_RM_ cells derived from nonepithelial tissues such as the kidney, adipose tissue, and liver [39, 40]. Thus, CD8^+^T_RM_ cells can classified into two subsets: CD103^−^CD8^+^T_RM_ and CD103^+^CD8^+^T_RM_. As compared with CD103^+^ CD8^+^T_RM_ cells, their CD103^−^T_RM_ counterparts display a greater propensity for transdifferentiation into other T cell subsets, indicating a more flexible but less stable residency. These CD103^−^T_RM_ cells also demonstrate increased proliferative potential, enhanced functionality, and reduced longevity relative to CD103^+^T_RM_ cells [39]. Moreover, the differentiation of CD8^+^T_RM_ cells in the small intestine and colon is regulated differently. Eomes has been found to repress the formation of CD8^+^T_RM_ cells in certain tissues while simultaneously promoting the maintenance of established CD8^+^T_RM_ cells in the small intestine, but not in the colon [117]. The diversity in phenotype and function allows CD8^+^T_RM_ cells to adapt effectively to local immune challenges, highlighting their importance in tissue‐specific immune responses.

CD8^+^T_RM_ cells exhibit not only intertissue heterogeneity, but also intratissue heterogeneity. In the skin, for example, CD8^+^T_RM_ cells are primarily identified by the expression of CD103, but they can be further categorized into two subsets based on CD49a expression: CD8^+^CD103^+^CD49a^+^ and CD8^+^CD103^+^CD49a^−^. These subsets differ in their compartmentalization and functional roles. The CD8^+^CD103^+^CD49a^+^ T_RM_ cells, referred to as T_RM_1, are found in human skin epithelia and are characterized by their ability to produce IFN‐γ. Upon stimulation with IL‐15, these cells express effector molecules such as perforin and granzyme B, indicating their cytotoxic potential. T_RM_1 cells are notably accumulated in both the epidermis and dermis of patients with vitiligo, suggesting their role in immune surveillance and response. Conversely, the CD8^+^CD103^+^CD49a^−^T_RM_ cells, identified in psoriasis lesions, are known as T_RM_17. These cells predominantly produce IL‐17, a cytokine associated with inflammation and autoimmunity, highlighting their involvement in pathogenic processes [71, 91]. In addition to the skin, the heterogeneity of CD8^+^T_RM_ cells has also been observed within the small intestine intraepithelial lymphocyte (siIEL) population in both mouse and human models [118, 119]. Within the lamina propria of the small intestine, two distinct subsets of CD8^+^T_RM_ cells have been characterized: CD8^+^CD103^+^ and CD8^+^CD103^−120^. CD103^+^CD8^+^T_RM_ cells rely on TGF‐β receptor signaling for their development, which underscores their importance in maintaining tissue integrity and homeostasis. In contrast, CD103^−^CD8^+^T_RM_ cells arise independently of TGF‐β signaling. Their differentiation is significantly influenced by inflammatory cytokines such as IL‐12 and IFN‐β, which are produced by macrophages during immune challenges [121]. This divergence in development leads to distinct functional roles for these subsets during immune responses. Spatial organization is another critical aspect of these CD8^+^T_RM_ cell subsets. CD103^−^CD8^+^T_RM_ cells often cluster with other immune cells at sites of bacterial infections, enhancing the localized immune response. Upon secondary infections, these cells can proliferate in situ, playing a vital role in controlling pathogens like *Yersinia pseudotuberculosis* [120, 122]. Conversely, while CD103^+^CD8^+^T cells show limited proliferation, they can arise from CD103^−^ precursors during reinfection, indicating a degree of plasticity and adaptability in their response to ongoing immune challenges [123]. Further investigations reveal that the TF STAT4 is essential for T_RM_ differentiation; its deficiency results in reduced numbers of intestinal CD103^−^CD8^+^T_RM_ cells by modulating TGF‐β‐driven expression of T_RM_ signature genes [124]. Additionally, the TF Hic1 can substitute for TGF‐β in promoting the formation of intestinal CD103^+^CD8^+^T_RM_ cells [125]. In human liver tissue, the composition of CD8^+^T_RM_ cells also reflects this heterogeneity, with approximately 95% being CD103^−^ and only about 5% expressing CD103 [126]. CD103^+^CD8^+^T_RM_ cells play a crucial role in the immune response against hepatotropic infections, exhibiting a robust expression of IL‐2, which positions them as key sentinels in the liver [127]. In contrast, CD103^−^CD8^+^T_RM_ cells are often described as bystander T cells. They display high expression of HIF‐2α and are implicated in the pathogenesis of various liver diseases [126]. Interestingly, similar subsets of CD8^+^T_RM_ cells are also found in the human cervix mucosa. In this context, CD69^lo^CD103^hi^CD8^+^T_RM_ cells are preferentially localized to the epithelial layer and are characterized by high expression of IFN‐γ, suggesting a potential role in antiviral responses. On the other hand, CD69^med^CD103^lo^CD8^+^T_RM_ cells are distributed more evenly between the epithelium and stroma and express high levels of Granzyme B, suggesting they may be involved in cytotoxic functions. Interestingly, there is little overlap in the TCR repertoires between these subgroups of CD8^+^T_RM_ cells, which implies distinct antigen specificities and functional roles within the tissue microenvironments [128]. This intratissue heterogeneity—both in location and function—highlights the importance of CD8^+^T_RM_ cells in anatomically specific immune responses.

Emerging evidence highlights the heterogeneity of CD8^+^T_RM_ cells in the course of their differentiation in response to infection and tumor. Studies have identified functionally distinct subsets of CD8^+^T_RM_ cells within the siIEL population at various stages of differentiation following viral infections [129]. For instance, effector‐like CD8^+^T_RM_ cells characterized as Blimp1^hi^Id3^lo^ are dominant in the early phase of infections. In contrast, memory‐like CD8^+^T_RM_ cells marked by Blimp1^lo^Id3^hi^ are tend to accumulate later in the infection process [130]. This differentiation heterogeneity of CD8^+^T_RM_ cells is also observed in malignant tissues, although they also exhibit features of Tex and Tpex cells [130]. Interestingly, while such heterogeneity in CD8^+^T_RM_ cell differentiation is present in healthy colonic tissue, conditions like ulcerative colitis can drive certain clonally related CD8^+^T_RM_ cells toward an inflammatory phenotype [131]. Overall, the dynamic differentiation and functional plasticity of CD8^+^T_RM_ cells underscore their critical roles in both protective immunity and potential pathological processes (Table 1).

**TABLE 1:** Phenotype and function heterogeneity of CD8^+^T_RM_ cells.

Classification	Tissue	Phenotype	Function	Refs.
Intertissue	Epithelial tissues	CD103^+^CD8^+^	Less flexible and more stable residency	[39, 40]
	nonepithelial tissues	CD103^−^CD8^+^	Increased proliferative potential, enhanced functionality, greater propensity for transdifferentiation and reduced longevity	[39, 40]
Intratissue	Human skin	CD103^+^CD8^+^CD49a+; CD103^+^CD8^+^ CD49a‐	CD8^+^CD103^+^CD49a^+^ T_RM_ are involved in vitiligo by production of cytotoxic effectors, while CD8^+^CD103^+^CD49a^−^ T_RM_ promote psoriasis by secretion of IL‐17	[71, 91]
	Intestine	CD103^−^CD8^+^; CD103^+^CD8^+^	CD103^+^CD8^+^ T cells show limited proliferation, they can arise from CD103^−^ precursors during reinfection; CD103^−^CD8^+^T_RM_ cells can rapidly proliferate and response to secondary infection	[118–120, 122]
	Human liver	CD103^−^CD8^+^; CD103^+^CD8^+^	CD103^+^CD8^+^T_RM_ cells play a crucial role in the immune response against hepatotropic infections, while CD103^−^CD8^+^T_RM_ cells are often described as bystander T cells and implicated in the pathogenesis of various liver diseases	[126, 127]
	Human cervix mucosa	CD69^lo^CD103^hi^CD8^+^; CD69^med^CD103^lo^ CD8^+^	CD69^lo^CD103^hi^CD8^+^T_RM_ play a potential role in antiviral responses by high expression of IFN‐γ, while CD69^med^CD103^lo^CD8^+^T_RM_ express high levels of Granzyme B, indicating cytotoxic functions.	[128]
Differentiation course	Infected tissue	Blimp1^hi^Id3^lo^; Blimp1^lo^Id3^hi^	Blimp1^hi^Id3^lo^ characterized as effector‐like CD8^+^T_RM_ cells are dominant in the early phase of infections; while memory‐like CD8^+^T_RM_ cells marked by Blimp1^lo^Id3^hi^ are tend to accumulate later in the infection process	[129, 130]
	Malignant tissue	Blimp1^hi^Id3^lo^; Blimp1^lo^Id3^hi^	Blimp1^hi^Id3^lo^ characterized as effector‐like CD8^+^T_RM_ cells also exhibit features of Tex; while Blimp1^lo^Id3^hi^ are memory‐like CD8^+^T_RM_ cells with Tpex features	[129, 130]

Abbreviations: CD8^+^T_RM_, tissue‐resident memory CD8^+^ T; HBV, hepatitis B virus; HDV, hepatitis D virus; MHCI, major histocompatibility complex class I; Tex, exhausted CD8^+^ T; Tpex, CD8^+^ progenitor exhausted T.

## 3 Biological Function of CD8^+^T_RM_

CD8^+^T_RM_ cells, first identified in infectious disease models, play a critical role in immune defense against pathogens at barrier sites and peripheral tissues. Notably, emerging evidence has also revealed an association between CD8^+^T_RM_ cells and favorable clinical outcomes in multiple cancer types. These findings have spurred significant research efforts to elucidate the biological functions of CD8^+^T_RM_ cells in combating infections and malignancies.

### 3.1 CD8^+^T_RM_ Against Reinfections

The role of CD8^+^T_RM_ cells in the immune response to pathogens is crucial for understanding how the body mounts an effective defense upon re‐exposure to previously encountered antigens. Unlike CD8^+^ T_CIRCM_ cells, which migrate between the bloodstream and lymphoid organs, CD8^+^T_RM_ cells are permanently stationed at sites of previous infections (e.g., mucosal surfaces, skin). This allows them to function as immediate responders to reinfection. Upon rechallenge with an antigen, CD8^+^T_RM_ cells can respond rapidly without the delay associated with the recruitment of CD8^+^T_CIRCM_ cells. CD8^+^T_RM_ cells recognize previously encountered antigens through TCRs^132^. This recognition is facilitated by antigen presentation, which can occur via monocyte‐derived DCs that are recruited to the site of infection [133]. The activation of CD8^+^T_RM_ cells can occur independently or dependently on CD4^+^T cell help [133, 134]. Once activated, CD8^+^T_RM_ cells can undergo homeostatic proliferation and employ various effector mechanisms to clear viral infections, primarily through the production of IFN‐γ and perforin‐mediated cytotoxicity [135]. In addition, the reactivated CD8^+^T_RM_ cells can also rapidly recruit CD8^+^T_CIRCM_ cells into peripheral tissues to synergistically combat the invading pathogens through secretion of IFN‐γ [132]. The interplay between CD8^+^T_RM_ cells and CD8^+^T_CIRCM_ cells might interpret why the relatively low numbers of CD8^+^T_RM_ cells in barrier tissues can still effectively control reinfections [80].

Studies on vaccinia virus demonstrate that localized infections in the skin can lead to the formation of CD8^+^T_RM_ cells not only at the site of infection but also in adjacent, noninvolved skin [134]. This is particularly important for rapid responses to reinfection or new infections that may occur in different areas of the skin. Intriguingly, unlike CD8^+^T_CIRCM_ cells, the reactivation CD8^+^T_RM_ cells in skin and female reproductive tract can also trigger protective innate immune responses, against antigenically unrelated viral infection [136, 137]. Therefore, CD8^+^T_RM_ cells can efficiently control cognate and antigenically unrelated pathogen infection by trigger of both protective innate and adaptive immune responses [136]. Thus, unlike CD8^+^T_CIRCM_ cells specialize in preventing systemic reinfection, CD8^+^T_RM_ cells mediate rapid immune responses for the efficient control of secondary infection at the local sites, while protecting the host from overt tissue damage [132, 136].

### 3.2 Central Role of CD8^+^T_RM_ in Tumor Immunity

Similar to their role in infection, tumor‐associated CD8^+^T_RM_ cells are essential for the immune surveillance of cancer cells. For instance, intradermal vaccination has been shown to promote the generation of skin‐resident CD8^+^T_RM_ cells, which provide protection against transplanted melanoma independent of CD8^+^T_CIRCM_ cells [138]. In the context of melanoma, CD8^+^T_RM_ cells generated during autoimmune vitiligo have been shown to protect against rechallenged melanoma, indicating that pre‐existing immune responses can effectively target tumor cells [139]. In addition, CD8^+^T_RM_ cells have also shown to actively survey dormant melanoma cells within the epidermis, and the depletion of CD8^+^T_RM_ cells promotes the development of epicutaneously transplanted melanoma in mouse skin, underscoring their role in local tumor immunity and surveillance [17]. The adoptively transfer of CD8^+^T cells lacking Runx3, which is vital for the differentiation of CD8^+^T_RM_ cells, leads to insufficient infiltration of CD8^+^ T cells into tumors and inadequate control of tumor growth [16].

CD8^+^T_RM_ cells characterized by the expression of CD103 have emerged as significant players in the immune landscape of various progressive tumors, influencing clinical outcomes. Notably, intraepithelial CD8^+^ tumor‐infiltrating lymphocytes (TILs) are associated with more favorable prognoses in epithelial cancers compared to their intrastromal counterparts [140]. Intraepithelial CD8^+^TILs, which exhibit high levels of CD103, interact with E‐cadherin on epithelial cells, a mechanism that may enhance their retention and effector functions within the tumor [18]. The density of these CD8^+^CD103^+^T cells has been linked to improved overall clinical outcomes across various epithelial tumors, including lung cancer [19], ovarian carcinoma [18], breast cancer [21], endometrial adenocarcinoma [22], urothelial cell carcinoma of the bladder [20], and HPV‐induced cervical cancer [23]. This suggests that the presence of these cells may play a critical role in antitumor immunity. Furthermore, intraepithelial CD8^+^TILs comprise both tumor‐specific and nonspecific CD8^+^T_RM_ cells. Interestingly, only the tumor‐specific CD8^+^T_RM_ cells upregulate the exhausted marker CD39 [141], allowing for the differentiation between these activated cells and bystander CD8^+^ T cells [41, 142]. The presence of tumor‐specific CD8^+^T_RM_‐like cells (CD39^+^CD8^+^T) has been associated with better overall survival in several solid tumors [24, 26, 142, 143], reinforcing the notion that targeting these specific immune populations could be a promising strategy for enhancing cancer immunotherapy.

CD8^+^T_RM_ cells play a crucial role not only in controlling tumor growth but also in preventing cancer metastasis. TdLNs are essential for priming antitumor immune responses, yet they paradoxically serve as common sites for early metastatic spread. Thus, the immune status of TdLNs is pivotal in determining the likelihood of tumor metastasis. Within TdLNs, CD8^+^T_RM_ cells have been identified, contributing to immune defense mechanisms, such as offering protection against infections like human immunodeficiency virus (HIV) [144]. Interestingly, while vaccination can induce tumor‐specific CD8^+^T_RM_ cells in lymph nodes [11, 145], this process can sometimes hinder immune responses against the primary tumor. This occurs through a reduction in the generation of CD8^+^ effector T cells and their migration from the lymph nodes into the tumor site [99]. On the other hand, tumor‐specific CD8^+^T_RM_ cells generated in contexts like melanoma‐associated vitiligo have been shown to provide long‐lasting protection against metastatic melanoma, correlating with improved patient prognosis [146]. Similarly, in human breast cancer, the presence of tumor‐specific CD8^+^T_RM_ cells—rather than the total CD8^+^T cell population—has been linked to delayed metastatic relapse following tumor resection, emphasizing their functional relevance in preventing metastasis [25]. Furthermore, the disruption of certain chemokine receptors, such as CXCR6 and CXCR16, has been associated with a reduction in tumor metastasis. This is thought to occur by promoting the formation of CD8^+^T_RM_ cells in distant tissues, thereby enhancing the immune landscape against metastatic spread [109]. Overall, bona fide CD8^+^T_RM_ cells and their T_RM_‐like counterparts are vital not only for preventing tumorigenesis but also for controlling tumor growth and regional metastasis. However, the role of CD8^+^T _RM_‐like cells in tumor control should be interpreted cautiously, as parabiosis assays [11] were not used to confirm their identity as bona fide CD8^+^T_RM_ cells (Figure 2B).

## 4 Cd8^+^T_RM_ and Diseases

CD8^+^T_RM_ cells play a critical role in protecting mucosal and barrier tissues against pathogen invasion. However, CD8^+^T_RM_‐like cells in the TME often exhibit exhaustion and functional impairment. Paradoxically, dysregulated activation of CD8^+^T_RM_ cells can also drive autoimmune disorders and chronic inflammatory diseases.

### 4.1 CD8^+^T_RM_ and Infectious Diseases

CD8^+^T_RM_ cells provide superior protection against pathogens that commonly infect mucosal and barrier tissues, such as the lungs, intestine, female reproductive tract and skin. In respiratory tract, CD8^+^T_RM_ cells are strategically located in areas like the nasal epithelial tissue of the upper respiratory tract and the pulmonary interstitium of the lower respiratory tract, where they can rapidly respond to infections by respiratory viruses, including IAV, respiratory syncytial virus (RSV), and severe acute respiratory syndrome coronavirus (SARS‐CoV) [5, 80, 147–152]. During infancy, the susceptibility to respiratory viral infections is partly attributed to the impaired establishment of lung CD8^+^T_RM_ cells, which limits the immune system's ability to mount effective responses [60]. Similarly, in aged individuals, the malfunction of CD8^+^T_RM_ cells contributes to insufficient heterologous immunity against IAV, resulting in a reduced capacity to combat diverse viral challenges [153]. In contrast to the long‐term persistence of CD8^+^T_RM_ cells in the upper respiratory tract [80], pulmonary CD8^+^T_RM_ cells have a relatively short lifespan, averaging about 200 days in mouse models [154]. This transient nature is attributed to several factors, including the retrograde migration of these cells into the draining mediastinal lymph nodes [155], as well as the impaired survival of CD8^+^T_EM_ cells and their suboptimal conversion into CD8^+^T_RM_ cells [154]. The gradual attrition of IAV‐specific lung CD8^+^T_RM_ cells correlates closely with a decline in heterosubtypic immunity, which is critical for recognizing and responding to diverse viral strains [154]. This loss is likely exacerbated by the unique pulmonary microenvironment, which is characterized by high levels of cellular stress due to its oxygen‐rich yet nutrient‐deprived conditions [156]. While repeated antigen exposure can enhance the durability of IAV‐specific CD8^+^T_RM_ cells in the lung [157], an excessive immune response may lead to tissue damage, adversely affecting lung function and overall respiratory physiology [158]. This delicate balance highlights the need for a well‐regulated immune response to maintain effective protection against respiratory infections without compromising lung integrity.

In the skin of mice, CD8^+^T_RM_ cells play a key role in immune surveillance, patrolling the epithelial layers and rapidly responding to reinfections at sites previously exposed to pathogens [159, 160]. This rapid detection and response mechanism is crucial for effective protection against re‐encounters with pathogens, such as herpes virus, where similar CD8^+^T_RM_ cells have been identified in human skin, contributing to immune surveillance [161, 162, 163]. In the gastrointestinal tract, oral infection with *Listeria monocytogenes* elicits a strong CD8^+^T_RM_ cell response in the intestines, which is essential for providing protection against subsequent infections [49]. Interestingly, after oral infection with *Yersinia pseudotuberculosis*, two distinct populations of pathogen‐specific CD8^+^T_RM_ cells are observed: CD8^+^CD103^+^T_RM_ cells and CD8^+^CD103^−^T_RM_ cells. Research indicates that while both populations are formed [120], it is the CD8^+^CD103^−^T_RM_ cells that primarily respond during secondary infections, suggesting a specialized role for this subset in mediating protective immunity [122].

Beyond barrier tissues, CD8^+^T_RM_ cells in organs such as the liver, kidney, and brain also play significant roles in protecting against infections. For instance, intranasal infection with vesicular stomatitis virus (VSV) or DC immunization can induce the generation of CD8^+^T_RM_ cells within the mouse brain [9]. Interestingly, the induction of these brain CD8^+^T_RM_ cells has been shown to be dispensable for local infections, as peripheral infections alone can sufficiently stimulate their generation against various pathogens, including *Toxoplasma gondii*, West Nile virus, LCMV, and VSV [164, 165, 166]. Upon reinfection, brain CD8^+^T_RM_ cells can quickly acquire cytotoxic effector functions, which are dependent on cognate antigen presentation. This enables them to prevent severe brain infections even in the absence of CD8^+^T_CIRCM_ cells [58, 135]. Moreover, evidence of CD8^+^T_RM_ has also been found in the human brain, displaying markers associated with T_RM_ cells. However, similar to their lung counterparts, human brain CD8^+^T_RM_ cells express relatively high levels of negative costimulatory molecules like PD‐1 and CTLA‐4, indicating that their immune surveillance activities against neurotropic infections are tightly regulated [167]. In the liver, CD8^+^T_RM_ cells are induced by the immunization with attenuated sporozoites and serve as a frontline defense against malaria sporozoite challenges [13]. Notably, CD103^+^CD8^+^T_RM_ cells are preferentially expanded in patients with partial immune control of hepatitis B virus (HBV) infection, and they can persist in the liver even after the resolution of the infection [127]. This suggests a sentinel role for CD8^+^T_RM_ cells in hepatotropic infections, emphasizing their importance in long‐term immune surveillance and protection across various organ systems.

### 4.2 Tumors Drive the Exhaustion and Dysfunction of CD8^+^T_RM_‐Like Cells

In tumors, CD8^+^ T cells that are generated in TdLNs can differentiate into Tpex cells, which then migrate into the TME and work to eliminate cancer cells [36]. However, chronic stimulation of TCR, along with constraints such as nutrient scarcity and low oxygen levels characteristic of solid tumors, can lead to the differentiation of Tpex cells into Tex cells. These Tex cells often lose their ability to proliferate and secrete effector cytokines, diminishing their antitumor efficacy [168]. Simultaneously, CD8^+^T_RM_‐like cells present in the tumor also display signs of exhaustion by upregulating immune inhibitory receptors such as PD‐1 and CD39, alongside a diminished capacity to produce essential effector cytokines like IL‐2 and TNF‐α [20, 23–28].

In the context of epithelial ovarian cancers, a distinct population of CD8^+^T_RM_‐like stem cells has been identified in the tumor epithelial region, characterized by markers such as TCF‐1^low^CD103^+^CD69^+ 169^. These CD8^+^T_RM_ stem cells are predictive of patient outcomes in ovarian cancer. However, these CD8^+^T_RM_ stem cells can further differentiate into various subsets, including effector, proliferative, and exhausted T_RM_ cells [169]. Both chronic TCR stimulation and the TME contribute to the dysfunction or exhaustion of CD8^+^T_RM_ cells, but the precise molecular mechanisms underlying these processes remain to be fully elucidated (Figure 2).

Of note, some key markers are overlapped between CD8^+^T_RM_‐like cells and Tex cells, including low expression of TCF1 [62, 63] and elevated expression of exhaustion marker TOX [129]. However, unlike Tex cells, tumor CD8^+^T_RM_‐like cells always highly express the cytotoxic, effector and proliferative genes [27, 169–172], and can persist in the skin of survivors of melanoma for a long time [173]. Thus, CD8^+^T_RM_ cells are distinct from Tex cells and exhibit enhanced functionality and longevity. However, it is a challenge to definitively distinguish genuine tumor‐reactive CD8^+^T_RM_ cells from Tex cells in the TME.

### 4.3 CD8^+^T_RM_ and Other Diseases

In addition to their critical roles in combating infections and tumors, CD8^+^T_RM_ are also implicated in the pathogenesis of autoimmune diseases, inflammatory disorders, and graft rejection following transplantation.

In the skin, CD8^+^T_RM_ are closely associated with the recurrence of inflammatory disorders [174]. Notably, the relapse of chronic cutaneous inflammation often occurs at previously resolved sites, underscoring the role of immunological memory in these processes. The long‐term persistence of CD8^+^T_RM_ cells in the epidermis suggests that they may be actively involved in the recurrence of inflammatory skin conditions. For instance, CD8^+^T_RM_ cells have been found to be enriched in the lesions of human vitiligo [69, 71, 91, 175], a chronic autoimmune depigmenting skin disorder. In vitiligo, The activation of T_RM_1, which exhibit the expression of effector molecules such as perforin and granzyme B upon stimulation with IL‐15, promotes a robust cytotoxic response, contributing to the pathogenesis of the disease [71]. Additionally, another study has demonstrated that CD8^+^T_RM_ cells in the skin of individuals with vitiligo express the chemokine receptor CXCR3, which is associated with migration to inflamed tissues. These CD8^+^T_RM_ cells also exhibit moderate cytotoxic activity, producing proinflammatory cytokines like IFN‐γ and TNF‐α, which further underscores their role in driving inflammation and tissue damage in autoimmune conditions [175]. CD8^+^T_RM_ have also been shown to cooperate with CD8^+^T_CIRCM_ cells to induce vitiligo in mouse [176]. This interaction highlights the complex immunological dynamics that contribute to the pathogenesis of vitiligo. The development of psoriasis, another autoimmune disorder characterized by chronic skin lesions and relapses, is also closely associated with the accumulation of CD8^+^T_RM_ cells [71, 91]. In patients with psoriasis, the activation of CD8^+^T_RM_ cells in nonlesional skin can trigger psoriasis‐associated tissue responses, indicating that even skin areas without visible lesions may harbor immune mechanisms ready to respond [177]. Notably, when symptomless prepsoriatic human skin is engrafted onto severe combined immune deficiency (SCID) mice, psoriasis can spontaneously develop, further emphasizing the role of these memory T cells in the disease process [178]. CD8^+^T_RM_ cells in psoriasis are characterized as T_RM_17, differing from the T_RM_1 phenotype seen in vitiligo [71]. The T_RM_17 exhibit a preference for secreting the inflammatory cytokine IL‐17, which is pivotal in driving psoriasiform responses and inflammation associated with psoriasis [91]. Research has indicated that the formation of T_RM_1 cells is regulated by IL‐15, while IL‐7 promotes the generation of T_RM_17 cells [91]. These insights suggest that targeting these cytokines may provide therapeutic strategies for treating autoimmune skin disorders like psoriasis and vitiligo. For example, antibody blockade of IL‐15 signaling has shown promise in durably reversing vitiligo [179], indicating that modulation of T_RM_ cell activity through cytokine targeting could be an effective approach in managing these conditions.

In the kidney, CD8^+^T_RM_ have been shown to significantly increase in both patients and mouse models with glomerular diseases [180, 181]. The administration of IL‐15 has been identified as a factor that promotes the formation and activation of CD8^+^T_RM_ cells, which in turn enhances podocyte injury and contributes to glomerulosclerosis [181]. This suggests that CD8^+^T_RM_ cells play a detrimental role in the progression of kidney diseases by exacerbating inflammation and tissue damage. In the liver, the frequency of CD103^+^CD8^+^T_RM_ cells correlates with the severity of autoimmune hepatitis. Treatment with glucocorticoids has been found to attenuate hepatic inflammation by directly inhibiting the expansion of these CD103^+^CD8^+^T_RM_ cells, indicating a potential therapeutic avenue for managing autoimmune liver diseases [182]. Furthermore, bystander CD8^+^T_RM_ cells, also classified as CD103^−^CD8^+^T_RM_ cells, have been significantly increased in patients with acute hepatitis A or cirrhosis [126]. These cells contribute to the pathogenesis of chronic hepatitis D virus (HDV) infection [183] and nonalcoholic steatohepatitis (NASH), possibly through mechanisms that are independent of major histocompatibility complex class I (MHCI) interactions [184]. Interestingly, while CD103^−^CD8^+^T_RM_ cells are implicated in promoting liver inflammation and disease, they also have a role in the resolution of liver fibrosis. They can induce apoptosis in hepatic stellate cells, which are key players in fibrosis development [185]. This dual role underscores the complexity of CD103^−^CD8^+^T_RM_ cell functions in liver pathology, where they can both contribute to disease progression and facilitate recovery.

CD8^+^T_RM_ cells also play a significant role in graft rejection following organ transplantation, a critical concern in the management of patients with end‐stage diseases. In kidney transplantation, graft failure can occur when recipient‐derived CD8^+^T_RM_ cells replace the donor‐derived CD8^+^T_RM_ cells within the transplanted kidney. These recipient‐derived CD8^+^T_RM_ cells can proliferate locally and produce proinflammatory cytokines such as IFN‐γ upon restimulation, which leads to damage of the donor kidney and contribute to acute and chronic rejection processes [187, 188]. In lung transplantation, a study have shown that recipients who maintain a higher number of donor‐derived lung CD8^+^T_RM_ cells experience fewer adverse events compared to those with lower persistence of donor CD8^+^T_RM_ cells. This suggests that the presence of donor‐derived CD8^+^T_RM_ cells may be beneficial for graft acceptance and overall transplant outcomes [186]. Therefore, approaches to improve organ transplant outcomes can include enhancing the persistence of donor‐derived CD8^+^T_RM_ cells to promote tolerance or finding ways to modulate recipient‐derived CD8^+^T_RM_ responses to prevent rejection (Table 2).

**TABLE 2:** The role of CD8^+^T_RM_ cells in autoimmune diseases, inflammatory disorders, and graft rejection.

**Classification**	**Disease**	**Mechanism**	**Refs**.
Autoimmune diseases	Psoriasis	T_RM_1 elicits a robust cytotoxic response to kill melanoma cells	[71, 175]
	Vitiligo	T_RM_17 promotes psoriasiform responses and inflammation associated with psoriasis by secreting IL‐17	[71, 91]
	Autoimmune hepatitis	Treatment with glucocorticoids attenuates hepatic inflammation by directly inhibiting the expansion of these CD103^+^CD8^+^T_RM_ cells	[182]
Inflammatory disorders	NASH	CD103^−^CD8^+^T_RM_ cells destroy hepatocytes independent on MHCI interaction	[184]
	Glomerulosclerosis	CD8^+^T_RM_ cells enhance podocyte injury and contributes to glomerulosclerosis	[180, 181]
	HDV/HBV/cirrhosis	The pathogenesis of chronic HDV, acute HAV and cirrhosis is correlated with CD103^−^CD8^+^T_RM_ cells, which destroy hepatocytes independent of MHCI interaction. However, they also have a role in the resolution of liver fibrosis	[126, 183]
Graft rejection	Kidney	Recipient‐derived CD8^+^T_RM_ cells replace the donor‐derived CD8^+^T_RM_ cells	[189, 190]
	Lung	Recipients with high level of donor‐derived lung CD8^+^T_RM_ cells experience fewer adverse	[186]

Abbreviations: HBV, hepatitis B virus; HDV, hepatitis D virus; ICI‐colitis, immune check inhibitor‐colitis; MHCI, major histocompatibility complex class I; NASH, nonalcoholic steatohepatitis.

## 5 Cd8^+^T_RM_ Cells‐Based Therapy

Building on the critical role of CD8^+^T_RM_ cells in combating infectious diseases, novel vaccine strategies have been designed to induce CD8^+^T_RM_ cell responses at mucosal and barrier sites. Conversely, in tumors, CD8^+^T_RM_‐like cells are frequently driven into exhaustion and dysfunction within the TME. To address this, emerging immunotherapies aim to reinvigorate CD8^+^T_RM_‐like cells to enhance antitumor immunity.

### 5.1 Vaccines Are Being Designed to Elicit a CD8^+^T_RM_ Cell Response Against Infectious Diseases

Given their ability to provide robust protection against pathogens that typically infect the skin, respiratory mucosa, and other tissues, designing vaccines based on CD8^+^T_RM_ presents a promising strategy for preventing infections at these entry sites [15].

#### 5.1.1 Immunization Strategies to Induce CD8^+^T_RM_ Cell Responses

The systematic divergent differentiation theory of CD8^+^T_RM_ cells outlines innovative vaccination strategies aimed at enhancing immune responses. These strategies include priming/pull [6, 189, 190], priming/trap [191], and priming/target [192] approaches, which consist of two key phases: initial vaccination to generate systemic T‐cell responses (the priming phase) and a subsequent immunization that facilitates the recruitment of activated CD8^+^T cells to specific tissues (the pull, trap, or target phase). In the first phase, a vaccination elicits a broad systemic immune response, generating a pool of activated CD8^+^T cells. In the second phase, these T cells are directed to tissues where they can differentiate into CD8^+^T_RM_ cells. This recruitment can be mediated by the expression of antigens or the release of inflammatory cytokines in the target tissues. For example, one study demonstrated that subcutaneous immunization with an attenuated HSV‐2 strain, combined with the topical application of chemokines CXCL9 and CXCL10 in the vaginal cavity of immunized mice, significantly increased the generation of HSV‐specific CD8^+^T_RM_ cells in the vagina [6]. In another context, to generate robust liver CD8^+^T_RM_ cell responses against malaria, naïve CD8^+^T cells are firstly activated by intramuscular immunization using recombinant viral vectors or DNA expressing malaria protective epitopes, then primed CD8^+^T cells are recruited to the liver through methods such as intravenous infection with a recombinant adeno‐associated virus vector (target) or by using attenuated sporozoites (trap) [191, 192].

The optimization of vaccination strategies for generating CD8^+^T_RM_ cells involves enhancing both the priming and pull/target/trap stages. Given that CD8^+^T_RM_ cells primarily differentiate from CD8^+^T_EF_ cells, the initial activation of these CD8^+^T_EF_ cells during the priming stage is critical for determining the subsequent quality and quantity of CD8^+^T_RM_ cells produced. A key factor in this process is the role of cross‐priming by DCs, particularly the subset characterized by DGNR‐1^+^ (CLEC9A^+^) markers. To enhance the efficiency of this priming phase, an innovative approach involves fusing anti‐Clec9A antibodies with malaria‐specific epitopes. This strategy aims to boost the priming efficiency of liver CD8^+^T_RM_ precursors, which can then be effectively recruited to the liver. Subsequent intravenous immunization with a recombinant adeno‐associated virus vector that expresses the corresponding malaria antigen facilitates the conversion of these precursors into functional CD8^+^T_RM_ cells [13]. At the pull/target/trap stage of the immunization process, it is crucial to establish an appropriate antigen expression and inflammatory microenvironment within the target tissues, facilitating the differentiation of the recruited CD8^+^T_EF_ into CD8^+^T_RM_ cells. Research indicates that local tissue vaccination is often more effective than systemic vaccination for this purpose. For instance, local intranasal immunization has been shown to successfully induce pulmonary CD8^+^T_RM_ cells, providing robust protection against IAV and *Mycobacterium tuberculosis*. In contrast, systemic or parenteral immunizations typically did not yield similar levels of CD8^+^T_RM_ cell generation in these tissues [193, 194].

The emerging evidence you mentioned highlights the significance of inducing CD8^+^T_RM_ through localized immunization strategies. For example, the intranasal administration of various vaccine modalities, including RSV antigen‐expressing vectors and COVID‐19 mRNA vaccines [148, 195–198], has demonstrated the ability to induce CD8^+^T_RM_ responses. These findings underscore the potential for intranasal vaccination not only to provide immediate protection but also to establish long‐lasting immune memory within the mucosal tissues, which are often the first line of defense against respiratory infections. Similarly, a single dose of glycolipid–peptide conjugate vaccine or mRNA vaccine immunized intravenously has also demonstrated significant efficacy in generating large populations of liver CD8^+^T_RM_ cells to prevent malaria infection [199]. The research also indicate that repeated exposure to antigens can enhance the expansion and durability of CD8^+^T_RM_ populations [157], which might be attribute to the local proliferation of existing CD8^+^T_RM_ cells, alongside the conversion of recruited CD8^+^T_CIRCM_ into CD8^+^T_RM_ [200, 201]. However, the ability of CD8^+^T_CM_ and CD8^+^T_EM_ cells to differentiate into CD8^+^T_RM_ cells appears to be context‐dependent, influenced by the specific tissue microenvironment [202, 203]. Overall, enhancing CD8^+^T_RM_ responses through extended immunization durations or persistent local antigen exposure could be a promising avenue for improving vaccine efficacy, particularly in the context of diseases where long‐term immunity is critical [204].

#### 5.1.2 Deliver Systems to Promote CD8^+^T_RM_ Cell Responses

Nanoparticles are increasingly recognized as an effective vaccine delivery system due to their ability to encapsulate antigens and provide a controlled, sustained release. This prolonged stimulation of the immune system is particularly advantageous for generating robust and enduring immune responses. For instance, the use of pH‐responsive nanoparticle vaccines administered at mucosal sites has been shown to induce protective CD8^+^T_RM_ cells in the lungs, highlighting the potential of nanoparticles to enhance local immunity [205]. Among the various nanoparticle platforms, lipid nanoparticles (LNPs) have emerged as a leading choice for developing vaccines against a wide range of infectious diseases [206]. One of the notable advantages of mRNA delivered via LNPs is its efficient targeting and expression in the liver when administered intravenously [207]. This mechanism has been pivotal in the development of mRNA‐based vaccines, which have demonstrated remarkable efficacy. For example, vaccines utilizing mRNA technology have successfully induced sterile protection against sporozoite challenges, showcasing their potential in combating diseases like malaria [208, 209]. Furthermore, the protective immunity elicited by mRNA COVID‐19 vaccines, such as the Pfizer‐BioNTech vaccine, has been associated with the expansion of CD8^+^T_RM_ cells in the nasal mucosa. This indicates that mRNA vaccines not only generate systemic immunity but also promote localized immune responses [198].

#### 5.1.3 Appropriate Adjuvant to Enhance CD8^+^T_RM_ Cell Responses

**FIGURE 3:**
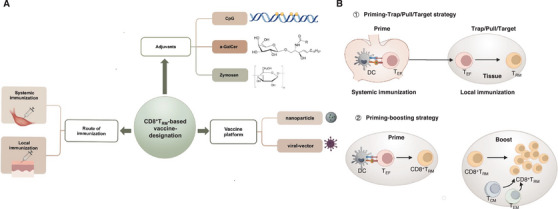
Vaccine strategy to induce CD8^+^T_RM_ cell response against infectious diseases. (A) The designation of CD8^+^T_RM_‐based vaccines can be optimized from three approaches. One is the choose of vaccine platform, such as nanoparticle and viral vector, in which the release of antigen is controlled and sustained; another is to choose appropriate adjuvant, such as TLR9 agonist CpG oligodeoxynucleotides (ODN), NKT cell agonist α‐galactosylceramide (α‐GalCer), and yeast cell walls component zymosan, to promote the generation of CD8^+^T_RM_ cells. Additionally, the route of immunization is also an important factor to optimize the CD8^+^T_RM_‐based vaccine designation. Although systemic immunization is used for priming CD8^+^T cells in priming‐trap/target/pull strategy, local immunization is prefer for single dose immunization strategy. (B) Two strategies have been proposed for the induction of CD8^+^T_RM_ cell response. One is prime‐trap/target/pull strategy. In this vaccination strategy, antigen‐specific CD8^+^T cells are first primed by systemically immunization of DNA vaccines, viral vector vaccines, or antigen‐targeted DCs, then the inflammatory status or antigen expression established by local immunization to trap/target/pull the activated CD8^+^T cells to the specific tissue. Another strategy is prime‐boost strategy. For this strategy, CD8^+^T cells are primed and differentiated into CD8^+^T_RM_ cells in tissue by local immunization, then CD8^+^T_RM_ cells are expanded by boosting immunization through local proliferation of CD8^+^T_RM_ cells, or generation of new CD8^+^T_RM_ cells differentiated from the recruited circulating memory CD8^+^T cells (CD8^+^T_CM_ or CD8^+^T_EM_).

Adjuvants also play a critical role in enhancing vaccine efficacy, particularly in eliciting CD8^+^T_RM_ cell responses. One of the most effective adjuvants identified is the TLR9 agonist CpG oligodeoxynucleotides (ODN), which has been shown to significantly promote the generation of liver CD8^+^T_RM_ cells when encapsulated in cationic liposomes [210]. Additionally, the NKT cell agonist α‐galactosylceramide (α‐GalCer) has demonstrated substantial effectiveness in inducing liver CD8^+^T_RM_ cell responses when conjugated with CD8^+^T cell antigenic peptides or encapsulated within LNPs [199, 208]. Another notable example is zymosan, an adjuvant derived from yeast cell walls, which has been shown to significantly boost lung CD8^+^T_RM_ development when coadministered intranasally with influenza vaccines [211]. However, the successful identification and development of appropriate adjuvants for enhancing CD8^+^T_RM_ cell responses will depend on a deeper understanding of the regulatory mechanisms governing the generation of these cells in various organs (Figure 3).

In summary, the successful induction of CD8^+^T_RM_ cell responses is influenced by multiple factors, including the vaccination strategy employed, the route of immunization, the choice of vaccine platform, and the formulation of adjuvants. By strategically manipulating these factors, it is possible to optimize CD8^+^T cell priming conditions and enhance tissue recruitment, thereby improving the lodgment of CD8^+^T_RM_ cells in peripheral tissues. This tailored approach could lead to more effective immune protection against various infections, ensuring a rapid and durable response upon re‐exposure to pathogens (Figure 3).

### 5.2 CD8^+^T_RM_‐Based Immune Therapy Against Tumors

#### 5.2.1 Tumor Vaccines and Chimeric Antigen Receptor T to Generate CD8^+^T_RM_

CD8^+^T_RM_ cells have emerged as crucial players in cancer immunosurveillance, recognizing and eliminating tumor cells in situ [16, 17, 212]. Their unique ability to persist within tissues and respond rapidly to tumor antigens makes them an attractive target for innovative immunotherapy strategies. Consequently, CD8^+^T_RM_‐based immunotherapies, including tumor vaccines and chimeric antigen receptor T (CAR‐T) cells, are being actively explored. Cancer vaccines designed to utilize polyacid microspheres or delivered via adenoviral vectors have shown promise in conferring protection against tumors. These vaccines effectively stimulate the generation of tumor‐specific CD8^+^T_RM_ cells, enhancing local immune responses against malignancies [213, 214]. Additionally, CAR‐T cell therapies are being refined to enhance their effectiveness in solid tumors. Recent study has demonstrated that CAR‐T_RM_ cells, generated in vitro through stimulation with TGF‐β, possess a heightened ability to infiltrate tumors. This infiltration is critical for their anticancer efficacy, as it allows these engineered T cells to directly engage with and eliminate cancer cells within the TME. The superior anticancer activity of CAR‐T_RM_ cells in solid tumors highlights the potential of combining CAR technology with strategies that promote the T_RM_ phenotype [215].

#### 5.2.2 The Response of CD8^+^T_RM_‐Like Cells in the TME to ICB Therapy

As mentioned above, CD8^+^T_RM_‐like cells always exhibit exhaustion and dysfunction in tumors, promoting they as promising targets for ICB therapy to enhance tumor‐specific CD8^+^T cell responses [20, 23–28]. Emerging evidence suggests that CD8^+^T_RM_ cells within TILs are responsive to ICB therapy, with their expansion being closely associated with the therapeutic efficacy of ICB treatments [26, 216–218]. This correlation highlights the importance of understanding the dynamics and functional roles of different CD8^+^T cell subsets in the context of ICB therapy, particularly in non–small‐cell lung cancer (NSCLC), where both CD8^+^T_RM_ and CD8^+^T_CIRCM_ have been identified [219]. Given the presence of both CD8^+^T_RM_ and CD8^+^T_CIRCM_ cells in NSCLC, it is crucial to evaluate the relative contributions of these subsets to the overall response to ICB therapy. Studies have shown that, compared to Tex cells, CD8^+^T_RM_ cells are often the primary responders to ICB therapy in various cancers, including hepatocellular carcinoma [220], head and neck squamous cell carcinoma [221], and triple‐negative breast cancer [222]. These findings challenge the previous notion that stem‐like tumor‐specific CD8^+^T cell subsets play the most critical role in ICB responses [36]. Interestingly, it is also possible that stem‐like CD8^+^T cell subsets may acquire T_RM_‐like features when exposed to the TME. Research has reported the presence of T_RM_ cells with stemness characteristics in human ovarian cancer, suggesting that these cells may be generated from stem‐like tumor‐specific CD8^+^T cells within tumors [169].

While the cytotoxic effects of CD8^+^T_RM_ cells against tumors have been highlighted in several studies [17, 30, 216, 222], the precise mechanisms through which these cells mediate antitumor immunity remain inadequately understood. Evidence suggests that CD8^+^T_RM_‐like cells can expand following ICB therapy, gaining the ability to effectively kill tumor cells. This cytotoxic capability is largely attributed to the high expression of cytotoxic mediators, such as granzyme B and perforin, which are crucial for inducing apoptosis in tumor cells [27, 30, 171]. Moreover, CD8^+^T_RM_ cells release inflammatory cytokines, including IFN‐γ and TNF‐α, which not only contribute to their direct antitumor effects but also help modulate the broader immune response [26, 220, 222]. In addition to their direct cytotoxic actions, CD8^+^T_RM_ cells may enhance antitumor immunity through their interaction with DCs in the draining lymph nodes. The cytokines secreted by CD8^+^T_RM_ cells can stimulate the maturation and cross‐presentation capabilities of DCs, thereby broadening the activation of cytotoxic CD8^+^T cell responses [28]. This mechanism is particularly significant as it links the resident memory response in the tissues with the adaptive immune response, amplifying the overall antitumor effect. Furthermore, the cytokines released by CD8^+^T_RM_ cells might also play a role in recruiting myeloid cells, such as neutrophils, to the tumor site [136]. Recent studies have indicated that the neutrophil response induced by ICB therapy correlates positively with favorable clinical outcomes in lung cancer patients [223]. This suggests that the interplay between CD8^+^T_RM_ cells and myeloid cell populations can enhance the overall effectiveness of the immune response against tumors.

**FIGURE 4:**
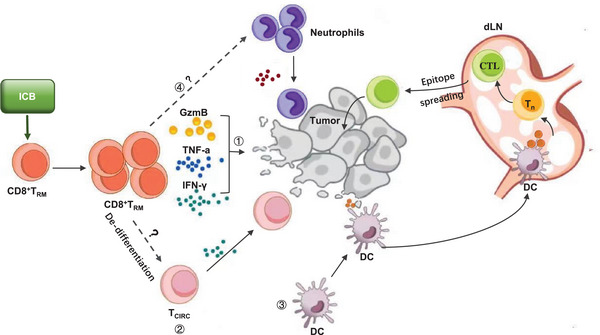
Dynamic response and killing mechanism of tumor‐specific CD8^+^T_RM_ after immune checkpoint blockade (ICB) therapy. After ICB therapy, tumor‐specific CD8^+^T_RM_ cells proliferate locally, and several mechanisms have been proposed for their ability to kill cancer cells. ① Tumor‐specific CD8^+^T_RM_ cells can directly destroy cancer cells through the production of cytotoxic molecules such as granzyme B, as well as cytokines like IFN‐γ and TNF‐α. ② CD8^+^T_RM_ cells may also indirectly kill tumor cells by dedifferentiating into circulating memory CD8^+^ T cells (Tcirc), which are then recruited to the tumor site by IFN‐γ secreted by the CD8^+^T_RM_ cells. ③ Cytokines secreted by CD8^+^T_RM_ cells can recruit DCs to the tumor microenvironment. These DCs can cross‐prime cytotoxic CD8^+^T cells against neoantigens and self‐antigens of the tumor in draining lymph nodes (dLNs), thereby amplifying the breadth of antitumor responses. ④ Additionally, cytokines released by CD8^+^T_RM_ cells might recruit neutrophils, further enhancing the efficacy of ICB therapy by targeting antigenically heterogeneous tumors. The symbol “?” indicates the unknown relationships between these compartments.

Recent findings suggest that infection‐generated CD8^+^T_RM_ cells maintain a degree of developmental plasticity, allowing them to dedifferentiate into T_EM_ cells [224]. In the context of cancer, study also has shown that oral cancer patients undergoing ICB therapy not only experience an expansion of local tumor‐specific CD8^+^T_RM_ cells but also an increase in activated T cells with the same clonotypes detected in the bloodstream [30]. This observation suggests that CD8^+^T_RM_ cells may dedifferentiate into circulating memory CD8^+^T cells during ICB treatment, contributing to a broader immune response against the tumor. Interestingly, the combination of TGF‐β blockade with ICB therapy has been shown to significantly reduce the retention of CD8^+^T_RM_ cells while increasing the frequency of circulating CD8^+^T cells. This combination therapy has been associated with improved clinical outcomes in patients, indicating that the interplay between CD8^+^T_RM_ cells and other tumor‐specific CD8^+^T cell subsets is crucial for orchestrating effective antitumor responses [225] (Figure 4).

However, it is important to consider that ICB therapy may induce overactivation of CD8^+^T_RM_ cells, potentially triggering autoimmune complications. For instance, ICI‐colitis—a common immune‐related adverse event of ICB therapy—has been closely linked to IFN‐γ‐producing CD8^+^T_RM_ cells [226, 227, 228].

## 6 Conclusion and Perspectives

CD8^+^T_RM_ cells are uniquely positioned to provide rapid and effective immune responses to reinfections at previously resolved sites within peripheral tissues. Their persistent localization allows for immediate recognition and response to pathogens, ensuring protection without causing significant tissue damage. This characteristic makes CD8^+^T_RM_ cells an attractive target for vaccine design, particularly for infections at barrier tissues such as the skin, lungs, and gastrointestinal tract. Interestingly, CD8^+^T_RM_‐like cells have also been identified in various solid tumors, where they are often associated with favorable clinical outcomes. However, in progressive cancers, these CD8^+^T_RM_‐like cells frequently exhibit exhaustion and dysfunction, highlighting their potential as novel targets for ICB therapy. Emerging evidence suggests that CD8^+^T_RM_‐like cells are among the predominant responders to ICB therapy, making them a focal point in efforts to enhance the efficacy of cancer treatments. However, it is crucial to be aware of the potential risks associated with the aberrant activation of CD8^+^T_RM_ cells. Therefore, successful therapies utilizing CD8^+^T_RM_ cells must be carefully designed to maximize their protective functions while minimizing the risk of adverse effects.

Despite growing evidence supporting the critical role of tumor‐specific CD8^+^T_RM_ cells in ICB therapy, several important aspects of these cells remain to be explored. First, the differentiation trajectory of tumor‐specific CD8^+^T_RM_ cells is still unclear. Second, it is crucial to confirm whether CD8^+^T cells with a residency phenotype in tumors are truly CD8^+^T_RM_ cells. Third, it is still unknown whether CD8^+^T_RM_ cells with exhaustion markers are simply exhausted CD8^+^ T cells that have gained tissue‐residency characteristics, or if the differentiated CD8^+^T_RM_ cells become exhausted within the TME. Finally, the dynamic response of tumor‐specific CD8^+^T_RM_ cells to ICB therapy, as well as the mechanisms by which these cells exert control over cancer, warrant further investigation.

CD8^+^T_RM_ cells exhibit key characteristics such as reduced expression of tissue‐egress proteins and heightened expression of tissue‐retention proteins. However, they also display significant heterogeneity in their transcriptional and epigenetic profiles across various tissues and disease states. This diversity in profiles enables a wide range of phenotypes and functional capabilities among CD8^+^T_RM_ cells, allowing them to adapt effectively to local immune challenges. Despite the importance of this heterogeneity, the mechanisms underlying the generation of CD8^+^T_RM_ diversity in different tissues and disease contexts remain largely unclear. This knowledge gap hampers the development of effective CD8^+^T_RM_‐based immunotherapies for infections and tumors. Therefore, it is crucial to investigate both the functional and phenotypic heterogeneity of CD8^+^T_RM_ cells in various tissues and disease states in future research.

## Author Contributions

Qizhao Huang and Lilin Ye conceived the structure of the manuscript. Luming Xu drafted the initial manuscript. Qizhao Huang and Lilin Ye revised the manuscript. Luming Xu prepared the figures. All the authors read and approved the final manuscript.

## Ethics Statement

Not applicable.

## Conflicts of Interest

The authors declare no conflicts of interest.

## Data Availability

The authors have nothing to report.
